# Racial Equity in Urine Drug Screening Policies in Labor and Delivery

**DOI:** 10.1001/jamanetworkopen.2025.0908

**Published:** 2025-03-17

**Authors:** Vahid Azimi, Cassandra Trammel, Lauren Nacke, Alexandra Rubin, Lori Stevenson, Brittaney Vaughn, Stephen M. Roper, Mark A. Zaydman, Ronald Jackups, Noor Riaz, Kim P. Schamel, Jeannie C. Kelly

**Affiliations:** 1 Department of Pathology and Immunology, Washington University School of Medicine in St Louis, St Louis, Missouri; 2 Department of Obstetrics and Gynecology, Washington University School of Medicine in St Louis, St Louis, Missouri; 3 Department of Pediatrics, Washington University School of Medicine in St Louis, St Louis, Missouri

## Abstract

**Importance:**

Black pregnant patients are significantly more likely than their White counterparts to undergo peripartum urine drug screening (UDS) and subsequent reporting to child protective services (CPS).

**Objective:**

To evaluate the association of removing isolated cannabis use and limited prenatal care as order indications, combined with clinician-facing clinical decision support, with racial parity in peripartum UDS and CPS reporting.

**Design, Setting, and Participants:**

This quality improvement study assessed 9396 pregnant patients at a single tertiary care center in a Midwestern US urban metropolitan region who delivered before (June 1, 2021, to September 31, 2022) and after (October 1, 2022, to January 31, 2024) the intervention.

**Exposure:**

Updated UDS indications combined with clinical decision support.

**Main Outcomes and Measures:**

Primary outcomes included UDS and CPS report rate by race before vs after the intervention. The secondary outcome was the rate of nonprescribed, noncannabis substance–positive UDS. Neonatal outcomes were included as balancing measures.

**Results:**

Of 9396 female patients (median [IQR] age, 29 [24-33] years; 4305 [45.8%] Black, 4277 [45.5%] White, and 814 [8.7%] other race) included in the analysis, 4639 and 4757 delivered in the preintervention and postintervention periods, respectively. There was a small but statistically significant decrease in the number of Black patients before vs after the intervention (2210 [47.6%] vs 2095 [44.0%], *P* = .005); there were no significant differences in other race groups, median age, or multiparity. Before the intervention, 513 (23.2%) and 228 (11.1%) Black and White patients, respectively, had UDS (*P* < .001) compared with 95 (4.5%) and 79 (3.6%) Black and White patients, respectively, after the intervention (*P* = .40). Before the intervention, an association between Black race and CPS report was observed (249 [11.3%] Black and 119 [5.8%] White patients, *P* < .001); there was no association between race and CPS report after the intervention (87 [4.2%] Black and 78 [3.5%] White patients, *P* = .67). There was no association between the intervention and the percentage of UDS results that were positive for nonprescribed, noncannabis substances (107 [2.5%] preintervention vs 88 [2.0%] postintervention; *P* = .14). There was no significant association between the intervention and any measured neonatal outcomes.

**Conclusions and Relevance:**

In this quality improvement study, removal of isolated cannabis use and limited prenatal care as UDS indications, coupled with clinical decision support, was associated with improved racial equity in UDS testing and CPS reporting. The intervention was not associated with a significant change in UDS positivity for nonprescribed, noncannabis substances. These findings suggest that this intervention improved equity in UDS practices without decreasing identification of clinically relevant substance use.

## Key Points

**Question:**

What is the association of removing isolated cannabis use and limited prenatal care as approved order indications for urine drug screening with racial parity in urine drug screening and reporting to child protective services in a hospital labor and delivery unit?

**Findings:**

In this quality improvement study of 9396 pregnant patients, updating the urine drug screening policy was associated with a significant reduction in racial disparities in testing and child welfare reports, with no change in balancing measures.

**Meaning:**

These findings suggest that an updated labor and delivery urine drug screening policy combined with clinical decision support may reduce racial bias in urine drug screening and reporting to child protective services without impacting the identification of clinically meaningful substance use.

## Teaser

This quality improvement study evaluates the association between removing isolated cannabis use and limited prenatal care as order indications for urine drug screening with racial parity in screening and reporting to child protective services.

## Introduction

Substance use disorder during pregnancy is common and associated with poor patient and newborn outcomes.^1^ The perinatal period offers an opportunity for behavioral intervention, and the American College of Obstetricians and Gynecologists (ACOG) recommends universal screening for substance use disorder in pregnancy via validated screening instruments to promote early identification and treatment.^2^ Laboratory drug tests, including urine drug screening (UDS), are often used for objective data about substance use in pregnancy^3^ to guide clinical management in specific clinical situations, such as altered mental status, unexplained hypertension, or monitoring of an exposed newborn. However, in the case of cannabis, which does not impact clinical care in the delivery admission, the benefit of testing is unclear.^4^

Conversely, UDS poses risk; in states such as Missouri, where our institution is located, any positive result for federally illegal or nonprescribed substances (including cannabis) during delivery must be reported to child protective services (CPS).^5^ Although CPS involvement may provide support, it also carries potential harms, such as criminalization, custody loss, or housing insecurity, which may deter patients from seeking care, worsening outcomes for both mother and newborn.^6,7^ Therefore, the potential benefits and harms of UDS should be carefully weighed before ordering.

Despite similar substance use disorder rates across racial and socioeconomic groups, Black and low-income patients are disproportionately subjected to UDS and its related harms.^8,9,10,11^ A previous study found that Black patients were significantly more likely than White patients to undergo UDS for isolated cannabis use and to be reported to CPS.^12^ Additionally, UDS for an indication of a history of isolated cannabis use alone had a poor positive predictive value for exposure to noncannabis substances, such as cocaine or opioids.^12^ Limited prenatal care (LPNC), a testing indication without clear definition, similarly targeted Black patients with low predictive value.^12^

In response, a multidisciplinary team developed a new protocol to enhance equity in UDS on our institutional labor unit. This protocol eliminated isolated cannabis use and LPNC as testing indications and introduced an electronic clinical decision support (CDS) tool requiring justification for UDS orders. We then sought to measure the association between these changes and racial parity in the rate of UDS ordering and subsequent reporting to CPS.

## Methods

In October 2022, a multidisciplinary committee composed of stakeholders from obstetrics, social work, neonatology, pediatrics, laboratory medicine, and nursing at our institution updated the protocol for UDS ordering on the labor unit to improve racial equity. Our institution is a level IV regional perinatal health care center that provides the highest level of obstetric and neonatal care in the region and is the larger of 2 hospitals that serve a predominately urban and historically underserved population in a midsized Midwestern city. Universal verbal drug screening is performed via a validated tool^13^; if screening is indicated, patients provide verbal consent for UDS. Perinatal UDS testing is performed by liquid chromatography–tandem mass spectrometry; technical details of the assay and a list of screened compounds are described in the study by Tesfazghi et al.^14^ First, isolated cannabis use was removed as an indication for UDS. Because the UDS panel is also used outside the labor setting, removal of cannabis from the panel was not within the scope of the committee, and masking cannabis results was impractical from a laboratory workflow and informatics standpoint. Second, LPNC was also removed as an indication for UDS. No prenatal care, defined as no care received during pregnancy at any institution, remained in the policy. The group concurrently implemented a mandatory electronic CDS tool in the order entry system that required the clinician to select an approved indication when ordering UDS (eTable 1 in Supplement 1). The combined order indication policy change and associated CDS will be referred to as the *intervention* throughout this article.

We performed a retrospective quality improvement study comparing UDS ordering and CPS reporting practices before (June 1, 2021, to September 31, 2022) and after (October 1, 2022, to January 31, 2024) the intervention. Because the electronic order question made it challenging for clinicians to order UDS without a policy-compliant indication, a washout period was not included. The institutional review board of Washington University School of Medicine in St Louis deemed this study quality improvement research and therefore exempt from review and informed consent. All deliveries and UDS results during these time frames were included in this study. The revised Standards for Quality Improvement Reporting Excellence (SQUIRE) reporting guidelines were followed during this investigation.^15^

Data on all UDS results and postintervention order indications were retrieved from the laboratory information system (Cerner Millennium), and data on patient demographics, CPS reports, delivery outcomes, substance exposure *International Statistical Classification of Diseases and Related Health Problems, Tenth Revision* (*ICD-10*) diagnosis codes, and order indications were retrieved from the electronic health record. Race, a social construct and not a biological determinant, was determined by self-report collected during clinical care in the electronic health record and included in the analysis because the primary goal of this study was to assess racial parity. Because Black and White patients represented more than 90% of the study cohort, we excluded patients of other races and ethnicities (American Indian or Alaska Native, Asian, Native Hawaiian or Other Pacific Islander, >1 race, or race not listed) from further analysis.

The primary outcomes were rates of UDS ordering and CPS reporting by race in the preintervention vs postintervention periods. UDS ordering and CPS report rates were calculated and reported as a proportion of both all deliveries and all tested patients during each period. Secondary outcomes included drug screen positivity rates for all delivery encounters and all tested patients, including isolated cannabis positivity, any noncannabis substance positivity, and any nonprescribed (ie, illicit), noncannabis substance positivity, as well as order indications in the postintervention group by race. Because we are unable to directly assess missed exposure diagnoses of neonates born to untested patients, outcome data on birth outcomes, neonatal intensive care unit admission, length of stay, and *ICD-10* codes associated with neonatal withdrawal (code P96.1: “Neonatal withdrawal from maternal use of drugs of addiction”) were included as balancing measures.

To investigate whether the policy change was associated with any change in UDS detection of nonprescribed, noncannabis substances, 2 authors (V.A. and C.T.) performed a secondary analysis with manual medical record review of all UDS results that were positive for noncannabis substances and excluded positive results that could be otherwise explained by a prescribed substance (eg, a positive fentanyl result that was associated with administration of an epidural). Nonprescribed substance positivity rates were then compared across the 2 time frames.

### Statistical Analysis

Hypothesis testing was performed using 2-sided χ^2^ and Mann-Whitney *U* tests as appropriate. The significance threshold was *P* < .05. *P* value correction for false discovery rate was performed by the Benjamini-Hochberg method. Statistical analysis and false discovery rate correction were performed using Python, version 3.9.6 (Python Software Foundation) and the Python SciPy and statsmodels packages, respectively.

## Results

In the 9396 female patients (median [IQR] age, 29 [24-33] years; 4305 [45.8%] Black, 4277 [45.5%] White, and 814 [8.7%] other) included in the analysis, there were 4639 and 4757 deliveries during the preintervention and postintervention periods, respectively. Black patients accounted for a slightly higher proportion of preintervention deliveries (2210 [47.6%] before and 2095 [44.0%] after the intervention; *P* = .005), whereas the number of White patients and patients of other races did not differ significantly. There were no significant associations between the intervention and other patient demographics, neonatal outcomes, or neonatal substance-related withdrawal diagnoses (Table 1).

**Table 1.** Maternal Characteristics and Neonatal Outcomes by Intervention Group

Characteristic or outcome	No. (%) of participants^a^		*P* value
	Preintervention period	Postintervention period	
**Maternal characteristics **			
No.	4639	4757	NA
Age, median, (IQR), y	29 (24-33)	29 (24-33)	.89
Race			
Black	2210 (47.6)	2095 (44.0)	.005
White	2054 (44.3)	2223 (46.7)	.25
Other^b^	375 (8.1)	439 (9.2)	.35
Multiparous	341 (7.4)	341 (7.2)	.89
**Neonatal outcomes**			
No.	4826	4948	NA
Preterm birth	1038 (21.5)	1040 (21.0)	.92
5-min Apgar score <7	330 (6.8)	381 (7.7)	.55
Birth weight <2500 g	981 (20.3)	996 (20.1)	.92
Birth weight <1500 g	277 (5.7)	273 (5.5)	.92
NICU admissions	598 (12.4)	577 (11.7)	.86
Length of stay, median (IQR), d	2 (1-2)	2 (1-2)	.15
Neonatal withdrawal *ICD-10* code	107 (2.3)	105 (2.2)	.97

Abbreviations: *ICD-10*, *International Statistical Classification of Diseases and Related Health Problems, Tenth Revision*; NA, not applicable; NICU, neonatal intensive care unit.

^a^ Unless otherwise indicated.

^b^ Other race includes American Indian or Alaska Native, Asian, Native Hawaiian or Other Pacific Islander, more than 1 race, or not listed.

There was a significant decrease in the proportion of patients undergoing UDS with the intervention (741 [17.4%] before vs 174 [4.0%] after the intervention, *P* < .001) (Table 2). A significant association between race and proportion of deliveries tested was observed before (513 [23.2%] Black and 228 [11.1%] White patients; *P* < .001) but not after (95 [4.5%] Black and 79 [3.6%] White patients; *P* = .40) the intervention (Table 3 and Figure). The intervention was associated with a significant decrease in isolated cannabis positivity per all deliveries (260 [6.1%] positive before and 19 [0.4%] positive after the intervention; *P* < .001) and any noncannabis substance positivity (220 [5.2%] positive before and 112 [2.6%] after the intervention; *P* < .001). However, after excluding prescribed substances via manual medical record review, the association between the intervention and nonprescribed, noncannabis substance was no longer statistically significant (107 [2.5%] positive before and 88 [2.0%] after the intervention; *P* = .14) (Table 2).

**Table 2.** UDS Testing, CPS Reporting, and UDS Positivity in the Preintervention vs Postintervention Period

Measure	No. (%) of participants		*P* value
	Preintervention period (n = 4264)	Postintervention period (n = 4318)	
UDS performed	741 (17.4)	174 (4.0)	<.001
CPS report	368 (8.6)	165 (3.8)	<.001
Any noncannabis substance positive UDS result	220 (5.2)	112 (2.6)	<.001
Nonprescribed, noncannabis substance positive UDS result	107 (2.5)	88 (2.0)	.14
Isolated cannabis positive UDS result	260 (6.1)	19 (0.4)	<.001

Abbreviations: CPS, child protective services; UDS, urine drug screen.

**Table 3.** UDS Testing, CPS Reporting, and UDS Positivity in the Preintervention vs Postintervention Period by Race

Variable	No. (%) of participants		*P* value
	Black (n = 4305)	White (n = 4277)	
UDS performed			
Preintervention	513 (23.2)	228 (11.1)	<.001
Postintervention	95 (4.5)	79 (3.6)	.40
CPS report			
Preintervention	249 (11.3)	119 (5.8)	<.001
Postintervention	87 (4.2)	78 (3.5)	.67
Any noncannabis substance positive UDS result			
Preintervention	115 (5.2)	105 (5.1)	.95
Postintervention	52 (2.5)	60 (2.7)	.92
Nonprescribed, noncannabis substance positive UDS result			
Preintervention	50 (2.3)	57 (2.8)	.84
Postintervention	43 (2.1)	45 (2.0)	>.99
Isolated cannabis positive UDS result			
Preintervention	210 (9.5)	50 (2.4)	<.001
Postintervention	16 (0.8)	3 (0.1)	.72

Abbreviations: CPS, child protective services; UDS, urine drug screen.

**Figure.**
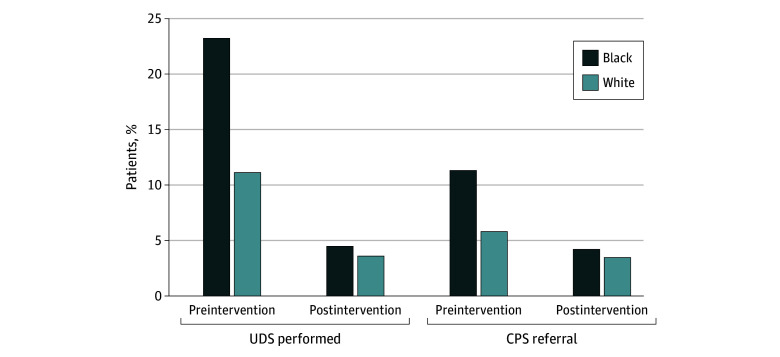
Urine Drug Screening (UDS) and Child Protective Service (CPS) Reporting by Race in the Preintervention and Postintervention Time Frames In the preintervention period, a larger proportion of Black patients underwent UDS (513 [23.2%] vs 228 [11.1%]; *P* < .001). In the postintervention period, there was no significant difference between groups (95 [4.5%] vs 79 [3.6%]; *P* = .4). In the preintervention period, a larger proportion of Black patients were referred to CPS than White patients (249 [11.3%] vs 119 [5.8%]; *P* < .001), whereas in the postintervention period, there was no significant difference between groups (87 [4.2%] vs 78 [3.5%]; *P* = .67).

Significant associations between race and isolated cannabis positivity were observed before the intervention (210 [9.5%] Black and 50 [2.4%] White; *P* < .001) but not after the intervention (16 [0.8%] Black and 3 [0.1%] White; *P* = .72). No differences were noted before vs after the intervention between race in noncannabis substance positivity (preintervention: 115 [5.2%] Black and 105 [5.1%] White [*P* = .95]; postintervention: 52 [2.5%] Black and 60 [2.7%] White [*P* = .92]) or nonprescribed, noncannabis substance positivity (preintervention: 50 [2.3%] Black and 57 [2.8%] White [*P* = .84]; postintervention: 43 [2.1%] Black and 45 [2.0%] White; *P* > .99) per delivery (Table 3).

When comparing positivity as the proportion of all patients tested, the intervention was associated with a significant increase in positivity for any noncannabis substance (220 [29.7%] before and 112 [64.4%] after the intervention; *P* < .001) (eTable 2 in Supplement 1) and any nonprescribed, noncannabis substance (107 [14.4%] before and 88 [50.6%] after the intervention; *P* < .001) (eTable 2 in Supplement 1) and a significant decrease in positivity for cannabis only (260 [35.1%] before and 19 [10.9%] after the intervention; *P* < .001) (eTable 2 in Supplement 1). Notably, there was a significant association in both periods between race and proportion of positive UDS results for any noncannabis substance (preintervention: 115 [22.4%] Black and 105 [46.1%] White [*P* < .001]; postintervention: 52 [54.7%] Black and 60 [75.9%] White [*P* < .001]) (eTable 2 in Supplement 1), nonprescribed, noncannabis substances (preintervention: 50 [9.7%] Black and 57 [25.0%] White [*P* < .001]; postintervention: 43 [45.3%] Black and 45 [57.0%] White [*P* < .001]) (eTable 2 in Supplement 1), and isolated cannabis positivity (preintervention: 210 [40.9%] Black and 50 [21.9%] White [*P* < .001]; postintervention: 16 [16.8%] Black and 3 [3.8%] White [*P* = .02]) (eTable 2 in Supplement 1) per all tested patients.

A significant association between the intervention and the proportion of delivering patients reported to CPS was observed (368 [8.6%] before and 165 [3.8%] after the intervention; *P* < .001) (Table 2). Importantly, a significant association between race and percentage of patients who were reported to CPS was observed before (249 [11.3%] Black and 119 [5.8%] White; *P* < .001) but not after (87 [4.2%] Black and 78 [3.5%] White; *P* = .67) the intervention (Table 3).

The most common UDS indication in the postintervention period was a “history of substance abuse, excluding cannabis” (69 [39.7%] of UDSs ordered). No association between UDS indications and race was observed (Table 4).

**Table 4.** Indications for Urine Drug Screens in the Postintervention Period by Race

Urine drug screen indication	No. (%) of participants		*P* value
	Black (n = 95)	White (n = 79)	
History of substance use, excluding cannabis	38 (40.0)	27 (34.1)	>.99
No prenatal care	13 (13.7)	20 (25.3)	.32
Opioids prescribed during pregnancy	8 (8.4)	6 (7.6)	>.99
Not applicable	1 (1.1)	6 (7.6)	.32
Unexplained late fetal demise or repeated spontaneous abortions	6 (6.3)	0	.32
Unexplained hypertensive crisis	7 (7.4)	4 (5.1)	>.99
Unexplained abruption of placenta	0	2 (2.5)	>.99
Sudden change in mental status	0	1 (1.3)	>.99
Unexplained seizure	1 (1.1)	0	>.99
Other (free text)			
No indication listed	10 (10.5)	9 (11.4)	>.99
Limited prenatal care	3 (3.2)	0	.48
Unexplained tachycardia	1 (1.1)	0	>.99
Intrauterine fetal demise	2 (2.1)	1 (1.3)	>.99
Elevated blood pressures	0	1 (1.3)	>.99
Placental abruption	1 (1.1)	0	>.99
History of substance use, excluding cannabis	2 (2.1)	2 (2.5)	>.99
27-wk Bleeding	1 (1.1)	0	>.99
Elevated liver function levels	1 (1.1)	0	>.99

## Discussion

This quality improvement study showed that implementing an evidence-based UDS policy with electronic CDS was associated with achievement of racial parity in testing and CPS reporting. The intervention was also associated with a decrease in overall UDS orders and isolated cannabis positivity. For nonprescribed, noncannabis substances, there was increased positivity among tested patients without a change among total deliveries. Finally, balancing measures did not reveal any significant association with neonatal outcomes.

The most significant outcome of our study was the association of the intervention with a decrease in the overall CPS reporting rate and racial parity among reports filed. We acknowledge that CPS referral may have safety benefit in some clinical situations and is certainly indicated in cases of child abuse; however, these authors would challenge the assumption that parental substance use, particularly of cannabis, which is legal in the state of Missouri, constitutes such a scenario. The literature on mandated reporting of positive UDS results demonstrates significant potential harms associated with this practice, including incarceration, loss of custody of the newborn, involuntary commitment, and loss of housing.^16^ A meta-synthesis evaluating the effectiveness of mandatory reporting found that most studies reported negative experiences, including accounts of harm to the patient-physician therapeutic relationship and child death after removal of custody from their family of origin. Because high-quality evidence on the effectiveness of mandatory reporting is lacking, it is critical to minimize the instances of this practice that are less likely to lead to any benefit for the patient and newborn, such as is the case for isolated cannabis use. Fear of mandated reporting is an often-cited concern among pregnant people, and in Missouri, where Black pregnant people are 4 times more likely to die within 1 year of pregnancy than White pregnant people, reducing barriers and increasing access to safe, excellent, and equitable care is paramount.^17^ Furthermore, if the goal of drug screening is to identify patients who would benefit from counseling and support, screening can be readily performed verbally with provision of resources in a clinical, rather than legal, setting.

Our study found that the intervention was associated with a greater than 75% reduction in overall UDS and improved racial parity. This reduction correlated with more than 2- and 3-fold increases in the percentage of tested patients testing positive for any noncannabis compound and nonprescribed, noncannabis compounds, respectively, and a 3-fold decrease in cannabis-only positive results. Because UDS aims to guide clinical management during delivery, these findings suggest the updated policy and clinical decision support significantly improved the positive predictive value of UDS for detecting nonprescribed, noncannabis compounds, enhancing its clinical utility. Although we cannot definitively assess changes in sensitivity for detecting nonprescribed substances, manual medical record review of noncannabis substance–positive UDS results suggested no change in sensitivity, as evidenced by stable rates of nonprescribed, noncannabis substance positivity per delivery.

Removing isolated cannabis use as an approved UDS order indication will naturally raise reasonable concerns about the impact of maternal cannabis use on the developing fetus. Cannabis is the second-most common (after alcohol) psychoactive substance used during pregnancy. Several observational studies have reported negative associations among fetal cannabis exposure, fetal development, neonatal outcomes, and neurocognitive development,^18^ and ACOG recommends that people who are pregnant or contemplating pregnancy should be encouraged to discontinue cannabis use.^4^ Although we recognize the potential harm of in utero exposure and agree with ACOG’s position, cannabis use during pregnancy in and of itself does not indicate that a person is potentially unfit to parent. Indeed, ACOG explicitly states that “seeking obstetric-gynecologic care should not expose a woman to criminal or civil penalties for marijuana use.”^4^ Supporting this argument is the finding that 196 of 197 patients (>99%) who tested positive for cannabis after undergoing UDS for isolated cannabis use in a previous study were discharged home with custody of the newborn,^12^ suggesting that this practice has low predictive value for identifying what CPS would deem unsafe for a newborn. Furthermore, isolated cannabis use is poorly predictive for other substances on UDS, further underscoring its limited value in obtaining clinically useful information.^12^ Lastly, a patient who met criteria for UDS based on isolated cannabis use would by definition have already disclosed cannabis use, thus enabling practitioners to counsel accordingly regarding risks of exposure.

Future studies should explore the generalizability of our findings, the clinical utility of including cannabis in UDS, and patient experiences with drug screening. Notably, the impact of removing isolated cannabis use and LPNC as UDS indications may vary based on the racial composition and drug use patterns of the patient population. Baseline analyses of racial parity in UDS ordering and indications are crucial before implementing interventions because racial disparities in cannabis positivity rates differ across institutions.^8,11,12^

We hope this intervention serves as a generalizable example of using the Joint Commission’s framework to mitigate health disparities.^19^ To replicate our approach, health care organizations can (1) monitor parity in outcomes by stratifying data by patient demographics, (2) investigate selection criteria causing biases, (3) evaluate the evidence supporting use of these criteria, (4) revise policies if criteria cause more harm than benefit, and (5) assess intervention impacts through ongoing monitoring. Applying this framework can help institutions reduce disparities and ensure more equitable care for all.

### Strengths and Limitations

Our study has several strengths. The electronic order question CDS allowed us to assess UDS indications, promote protocol adherence, and prospectively monitor equity. Using data from our laboratory information system and electronic health records, we identified all relevant deliveries, UDS results, and CPS reports without manual review, enabling accurate analysis of UDS positivity rates. A multidisciplinary committee, including experts in obstetrics, pediatrics, nursing, laboratory medicine, and informatics, ensured a comprehensive approach and facilitated practitioner support. Importantly, although prior studies^8,9,10,11^ report racial disparities in UDS, few describe interventions. Although Peterson et al^20^ associated a standardized UDS protocol with reduced racial disparities, to our knowledge, this is the first study showing that improving equity in UDS testing does not significantly impact sensitivity for detecting nonprescribed, noncannabis substances. Additionally, our intervention improved the positive predictive value for nonprescribed, noncannabis substances, mainly by reducing isolated cannabis positive results.

Our study also has several limitations. Because this is an observational study, we cannot rule out confounders, although no concurrent institutional programs aimed at health equity or UDS were identified. Additionally, some criteria in the updated UDS policy (eg, labile behavior) remain open to interpretation, potentially introducing clinician bias. Unfortunately, removing cannabis from the UDS panel, a simpler intervention reported by other institutions, was not feasible here, and this single-institution study may have limited generalizability to multihospital settings. Furthermore, we cannot isolate the individual impact of removing isolated cannabis use or LPNC as UDS order indications, although prior data suggest that isolated cannabis use was a primary driver of UDS reduction.^12^ After the intervention, a small fraction of UDS orders lacked indications or listed LPNC, but less than 1% of patients tested positive for cannabis only, minimizing concerns of loophole misuse of the order set. Additionally, due to our patient population’s racial composition, we could not assess the intervention’s impact in racial groups other than Black or White. We also lacked reliable socioeconomic data and could not directly evaluate neonatal outcomes, such as neonatal opioid withdrawal syndrome, although related *ICD-10* codes showed no significant postintervention changes. Finally, long-term maternal and child outcomes from reduced CPS referrals were beyond this study’s scope and warrant future research.

## Conclusions

In this quality improvement study of an intervention that included an evidence-based protocol that refined UDS indications and implemented electronic decision support to guide appropriate ordering, the intervention was associated with improved racial parity in testing and reporting, with no evidence of decreased identification of nonprescribed, noncannabis substances. UDS must balance clinical benefits, such as guiding delivery management, against potential harms, such as patient alienation and CPS involvement. Evidence does not support UDS for isolated cannabis use or LPNC, practices that disproportionately affect Black patients. We remain committed to evaluating our screening policies to maximize benefits and minimize harms for patients with substance use disorder and their newborns. Although recent policy changes reduced CPS reporting and racial disparities, nearly 4% of delivering patients still have CPS files opened. The impact of these reports on outcomes will guide future policies.

## Notes

Supplement 1. **eTable 1.** List of Available Approved Order Indications for Electronic Urine Drug Screen (UDS) Order **eTable 2.** UDS Positivity Pre- vs Post-intervention, Stratified by Black or White Race

Supplement 2.
Data Sharing Statement
