# Supplementary material for: Racial Equity in Urine Drug Screening Policies in Labor and Delivery
Source: JAMA Netw Open. 2025 Mar 17;8(3):e250908. doi: 10.1001/jamanetworkopen.2025.0908 (PMC11915058; doi:10.1001/jamanetworkopen.2025.0908)
Supplement: Supplement 1. — eTable 1. List of Available Approved Order Indications for Electronic Urine Drug Screen (UDS) Order eTable 2. UDS Positivity Pre- vs Post-intervention, Stratified by Black or White Race [file jamanetwopen-e250908-s001.pdf]

Supplemental Online Content

Azimi V, Trammel C, Nacke L, et al. Racial equity in urine drug screening policies in labor and delivery. *JAMA Netw Open*. Published online March 17, 2025. doi:10.1001/jamanetworkopen.2025.0908

eTable 1. List of Available Approved Order Indications for Electronic Urine Drug Screen (UDS) Order

eTable 2. UDS Positivity Pre- vs. Post-intervention, Stratified by Black or White Race

This supplemental material has been provided by the authors to give readers additional information about their work.

© 2025 Azimi V et al. *JAMA Network Open*.

eTable 1: List of approved order indications for electronic order question: "Please select the labor and delivery indication for ordering."
Does not apply because the patient is not on labor and delivery
Sudden change in mental status
Pinpoint or dilated pupils
Aggressive or labile behaviors
Unexplained late fetal demise or repeat spontaneous abortions
Unexplained abruption of placenta
Unexplained seizure
Unexplained hypertensive crisis
No prenatal care
Substance use during pregnancy, excluding marijuana
History of opioids prescribed during pregnancy
Other (requires free text comment)

© 2025 Azimi V et al. *JAMA Network Open*.

eTable 2: UDS Positivity Pre- vs. Post-intervention, Stratified by Black or White Race			
	**Pre-intervention (n=741)**	**Post-intervention (n=174)**	***P***
**UDS performed (% tested)**			
Black	513 (69.2)	95 (54.6)	<0.001
White	228 (30.8)	79 (45.4)	<0.001
**Any Non-THC Positive UDS (% tested)**			
Total	220 (29.7)	112 (64.4)	<0.001
Black	115 (22.4)	52 (54.7)	<0.001
White	105 (30.8)	60 (75.9)	<0.001
**Illicit Non-THC Positive UDS (% tested)**			
Total	107 (14.4)	88 (50.6)	<0.001
Black	50 (9.7)	43 (45.3)	<0.001
White	57 (25.0)	45 (57.0)	<0.001
**Isolated THC Positive UDS (% tested)**			
Total	260 (35.1)	19 (10.9)	<0.001
Black	210 (40.9)	16 (16.8)	<0.001
White	50 (21.9)	3 (3.8)	0.02
Abbreviations: UDS = urine drug screen<br>Percentage calculations represent the proportion for that specific racial subgroup			

© 2025 Azimi V et al. *JAMA Network Open*.
