# Supplementary material for: Racial Equity in Urine Drug Screening Policies in Labor and Delivery
Source: JAMA Netw Open. 2025 Mar 17;8(3):e250908. doi: 10.1001/jamanetworkopen.2025.0908 (PMC11915058; doi:10.1001/jamanetworkopen.2025.0908)
Supplement: Supplement 2. — Data Sharing Statement [file jamanetwopen-e250908-s002.pdf]

Data Sharing Statement

Azimi. Racial Equity in Urine Drug Screening Policies in Labor and Delivery. *JAMA Netw Open*. Published March 17, 2025. doi:10.1001/jamanetworkopen.2025.0908

Data
Data available: No

Additional Information
Explanation for why data not available: Data can be made available upon request.
